# Cephalosporin allergy: R1 side-chain and penicillin cross-reactivity patterns in an Australian cohort

**DOI:** 10.1016/j.jacig.2025.100583

**Published:** 2025-10-23

**Authors:** Brittany Stevenson, Elizabeth Klinken, Michelle Trevenen, Jack Bourke, Patricia Martinez, Michaela Lucas

**Affiliations:** a Department of Immunology, Fiona Stanley Hospital, Perth, Australia; b PathWest Laboratory Medicine, Perth, Australia; c Department of Immunology, Sir Charles Gairdner Hospital, Perth, Australia; d Centre for Applied Statistics, School of Physics, Mathematics and Computing, University of Western Australia, Perth, Australia; e School of Medicine, University of Western Australia, Perth, Australia

**Keywords:** Allergy, cephalosporin, cross-reactivity, penicillin

## Abstract

**Background:**

Cross-reactivity between cephalosporins, and penicillins, is mainly explained by R1 side-chain similarity. However, data on cross-reactivity patterns in cephalosporin-allergic patients, with and without preexisting penicillin allergy labels, are lacking.

**Objective:**

We sought to determine whether R1 side-chain groups account for cross-reactivity between cephalosporins, and penicillins with similar R groups, in a cohort of patients with cephalosporin allergy labels, with and without a penicillin allergy history.

**Methods:**

A retrospective audit (February 2016 to November 2021) of adult outpatients with cephalosporin allergy labels, who underwent skin prick / intradermal testing and/or oral provocation challenges, was performed at 2 Australian tertiary hospitals.

**Results:**

We identified 212 patients with a single cephalosporin allergy label; 97 had coexisting penicillin allergy labels. Fifty-eight (27.4%) patients were confirmed as allergic to the index cephalosporin (47 to cefazolin). The cephalosporin skin testing and oral provocation challenge results were adequately explained by R1 side-chain patterns. Most (87.5%) of the cephalosporin-allergic patients with penicillin allergy labels tolerated a penicillin challenge and were delabelled. However, positive penicillin skin test results were found in 5 (10.2%) cefazolin-allergic patients without preexisting penicillin allergy labels.

**Conclusions:**

R1 side-chain groups explained most test outcomes in this study. However, some positive penicillin skin testing results were identified without a history of penicillin allergy. Future research investigating the safety of supervised graded penicillin challenges in cephalosporin-allergic patients is needed.

## Introduction

Cephalosporins are commonly implicated in drug hypersensitivity reactions.^1^, ^2^, ^3^, ^4^ In Australia and the United States, 0.8% to 6.9% of patients report a cephalosporin allergy label (CAL) and cefazolin is the most frequently implicated antibiotic in perioperative anaphylaxis.^1^^,^^2^^,^^5^ Contemporary evidence has established the cephalosporin R1 side-chain as the dominant antigen explaining most cross-reactivities within cephalosporins, and with penicillins.^6^^,^^7^ This raises the question as to whether beta-lactam (BL) cross-reactivities exist outside R1 side-chain groups. Data on this issue are sparse. Most studies on cephalosporin allergies have excluded subjects with multiple cephalosporin and/or penicillin allergies; thus, these cannot comprehensively address this question.^7^^,^^8^

**Fig 1:**
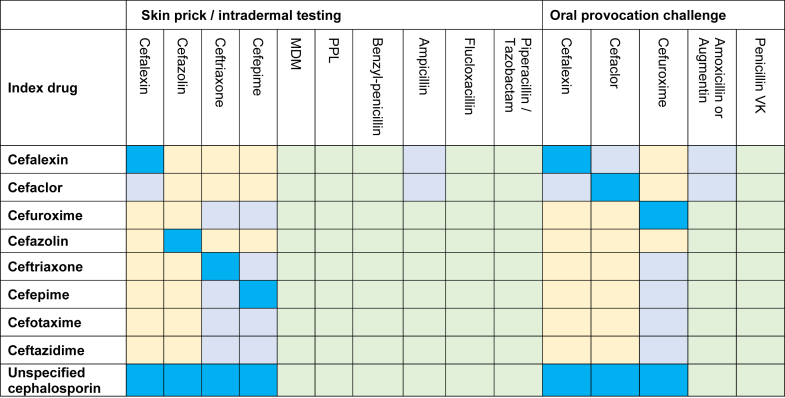
BL skin test and OPC antibiotics were categorized according to their R1 side-chain similarity. Bright blue, testing with index drug; pale blue, testing within the R1 side-chain group; yellow, testing with an unrelated cephalosporin; green, testing with an unrelated penicillin. Antibiotics with a similar, but nonidentical R1 side-chain have been grouped, that is, cefalexin and cefaclor with amoxycillin (similar R1 side-chain) and cefuroxime with cefepime, cefotaxime, ceftriaxone, and ceftazidime (nonidentical R1 side-chain, but some evidence of clinical cross-reactivity). *MDM*, Minor determinant mixture; *PPL*, penicilloyl polylysine.

A retrospective audit of adult subjects (≥16 years) with a CAL, including subjects with a coexisting penicillin allergy label (PAL) or multiple CALs, was conducted at 2 tertiary Western Australian drug hypersensitivity clinics between February 2016 and November 2021. During this study period, BL skin testing (ST) was available only at these 2 tertiary clinics. The morphology and chronology of the index reaction was assessed by a clinical immunologist, and subjects with definite non–IgE-mediated reactions, and subjects who declined testing, were excluded. Allergic reactions were graded according to the World Allergy Organization (WAO) Systemic Allergic Reaction Grading System, which, unlike other grading systems, classifies hypotension as grade 4.^9^ Subjects underwent ST with nonirritant skin prick and intradermal test concentrations with a panel of BLs, based on recommended ST concentration ranges in Australasian Society of Clinical Immunology and Allergy and other guidelines^10^^,^^11^ (Table I). Cephalosporin ST concentrations (1-2 mg/mL) were chosen from the suggested ST concentration ranges, based on local experience. Oral provocation challenges (OPCs) were performed as 2- or 3-dose graded challenges with a penicillin and/or cephalosporin selected by the drug allergy specialist. Intravenous challenges were not performed. Study aims were to (1) compare cephalosporin cross-/co-reactivity with predicted R1 side-chain patterns and (2) assess whether the R1 side-chain model was applicable in patients with PALs. Cephalosporins and aminopenicillins were categorized by R1 side-chain similarity on the basis of previous evidence (Fig 1).^12^ Approval was granted, with waiver of consent, by the relevant ethics boards (GEKO#40051 and GEKO#40317).

**Table I:** Skin prick and intradermal test concentrations

Skin prick test	
PPL	6.0 × 10^-5^ mol/L
MDM	1.5 × 10^-3^ mol/L
Benzylpenicillin	10,000 U/mL
Amoxicillin	20 mg/mL
Intradermal test	
Cefazolin	1 mg/mL
Ceftriaxone	1 mg/mL
Cefalexin∗	2 mg/mL
Cefepime	1 mg/mL
PPL	6.0 × 10^-5^ mol/L
MDM	1.5 × 10^-3^ mol/L
Benzylpenicillin	1,000 U/mL, 10,000 U/mL
Amoxicillin	20 mg/mL
Ampicillin∗	20 mg/mL
Flucloxacillin†	2 mg/mL
Piperacillin/tazobactam†	4.5 mg/mL

The BL panel was used for all subjects, with the exception of cefalexin, ampicillin, and piperacillin/tazobactam. MDM and PPL manufactured by Diater Laboratorio de Diagnostico, Madrid, Spain.

*MDM*, Minor determinant mixture; *PPL*, penicilloyl polylysine.

∗ Cefalexin test, and ampicillin intradermal test, was available for testing at only 1 hospital.

† Flucloxacillin was not always included, depending on clinician preference. Piperacillin/tazobactam was included if implicated in the index reaction.

## Results and discussion

Data were collected from 226 patient records: 212 subjects had a single CAL and 14 subjects had multiple CALs. Of the 212 subjects, 164 (77.4%) were female, mean age 50.5 years (range, 15-89 years). Implicated cephalosporins were cefalexin (n = 94 [44.3%]), cefazolin (n = 73 [34.4%]), ceftriaxone (n = 14 [6.6%]), cefaclor (n = 12 [5.7%]), cefepime (n = 7 [3.3%]), cefuroxime (n = 2 [0.9%]), cefotaxime (n = 1 [0.5%]), cetazidime (n = 1 [0.5%]), and “unspecified cephalosporin” (n = 8 [3.8%]). The grade of index reaction was mild (WAO grade 1-2) in 91 (42.0%) subjects, anaphylaxis (WAO grade 3-4) in 120 subjects (56.7%), and unknown in 1 (0.5%) subject. Time to testing was less than 1 year since index reaction in 86 (40.6%) subjects, 1 to 5 years in 55 (25.9%) subjects, and more than 5 years in 9 (4.3%) subjects. PALs were documented in 97 (45.8%) subjects, being most common with index reactions to cefalexin and cefaclor (62 of 106 [58.5%]).

**Fig 2:**
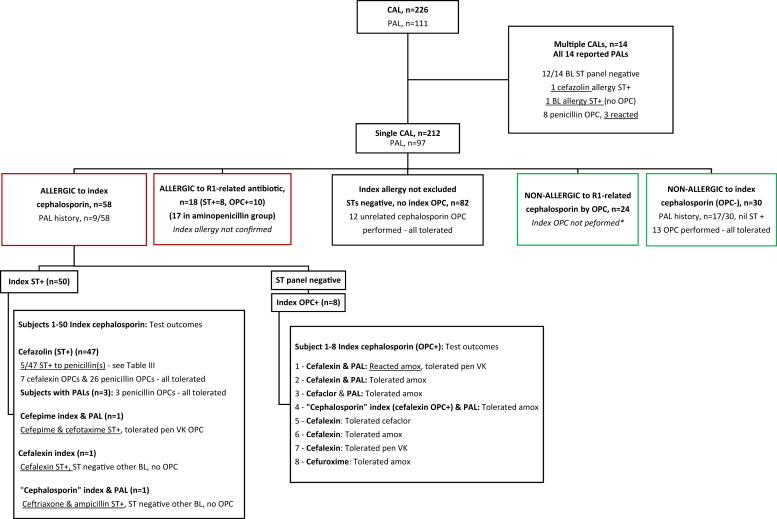
Skin prick / intradermal test and OPC outcomes for all subjects with CALs. Test outcomes relating to PALs are also summarized. *Amox*, Amoxicillin. ∗ST result negative, index OPC not performed for clinical or logistical reasons, noting intravenous challenges are not routinely performed.

Testing outcomes for the whole cohort are summarized in Fig 2. Overall, 58 (27.4%) subjects were confirmed allergic to the index cephalosporin by ST (n = 50) or OPC (n = 8). Forty-seven (81.0%) of the 58 subjects had an index reaction to cefazolin confirmed by ST, and 50 (86.2%) subjects had anaphylaxis as the index reaction (most due to intraoperative anaphylaxis). Nine (15.5%) of these 58 subjects had a previous PAL.

Multivariate logistic regression analysis was performed to assess predictors of positive ST results. Subjects with a history of anaphylaxis as the index reaction, or an intravenous culprit cephalosporin, were significantly more likely to have a positive ST result to the culprit or an R1 side-chain–related cephalosporin (index reaction anaphylaxis vs nonanaphylaxis: odds ratio, 6.05, 95% CI, 2.51-14.6, *P* < .0001; intravenous vs oral culprit cephalosporin: odds ratio, 9.68, 95% CI, 4.23-22.2, *P* < .0001) (Table II).

**Table II:** Univariate and multivariate logistic regression results comparing patient and index characteristics with ST result (event = positive index or R1 side-chain ST) and OPC (event = reacted to index or R1 side-chain OPC)

Characteristic	ST				OPC			
	Univariate		Multivariate		Univariate		Multivariate	
	OR (95% CI)	*P* value	OR (95% CI)	*P* value	OR (95% CI)	*P* value	OR (95% CI)	*P* value
Age								
For a 10-y increase	0.90 (0.76- 1.06)	.1861	Not significant		1.13 (0.81- 1.56)	.4837	Not significant	
Sex								
Male vs Female	2.22 (1.10- 4.48)	.0257	Not significant		1.29 (0.29- 5.71)	.7411	Not significant	
Grade								
3/4 vs 1/2/Unknown	6.69 (2.95- 15.2)	<.0001	6.05 (2.51- 14.6)	<.0001	1.11 (0.35- 3.49)	.8644	Not significant	
Timing								
Intermediate vs Remote/Unknown	1.13 (0.42- 3.06)	.0005	Not significant		1.43 (0.37- 5.46)	.8444	Not significant	
Recent vs Remote/Unknown	3.88 (1.74- 8.65)				1.37 (0.33- 5.75)			
Culprit route								
Intravenous vs Oral	10.4 (4.71- 23.1)	<.0001	9.68 (4.23- 22.2)	<.0001	1.47 (0.12- 17.4)	.7612	Not significant	
Culprit group								
Single CAL only vs single CAL + PAL	4.70 (2.25- 9.85)	<.0001	Not significant		0.59 (0.18- 1.98)	.3932	Not significant	

“Cephalosporin not specified” culprits excluded. No variables (age, sex, severity/timing of index reaction, drug route, presence of PAL) were statistically related to index or R1 side-chain OPC outcomes.

All 58 subjects with confirmed CALs underwent ST with a panel of BLs, followed by OPCs selected with the aim of safely broadening future BL options (Fig 2). R1 side-chain cross-reactivity was assessed in 11 subjects, excluding 47 subjects with cefazolin allergy due to its unique R1 side-chain. ST from 1 subject demonstrated R1 side-chain cephalosporin cross-reactivity (cefepime culprit and cefotaxime ST+). Six of the 11 subjects underwent an OPC with an R1-related antibiotic (all aminopenicillins), of which only 1 subject (cefalexin culprit) reacted with urticaria during an amoxicillin OPC.

We next assessed cross-reactivity for *un*related R1 side-chain cephalosporins. All 58 subjects had negative ST results to unrelated R1 side-chain cephalosporins. Seven subjects underwent an OPC with an unrelated cephalosporin, and all were tolerated.

Finally, we looked for examples of cross-/co-reactivity to penicillins or broad BL reactivity, excluding R1 side-chain–related penicillins (ie, aminopenicillins with aminocephalosporins). We compared patients with and without preexisting PALs. Of the 9 subjects with a preexisting PAL, 1 had a positive ST result to a penicillin (intradermal test cefazolin 7 mm, ampicillin 5 mm) and was not challenged; the other 8 subjects in fact tolerated a penicillin OPC and were delabelled. Thus, most (7 of 8 tested [87.5%]) of the subjects with confirmed CALs had their preexisting PAL removed. However, of the 49 subjects *without* any preexisting PAL, 5 subjects (10.2%) had positive ST results to penicillin(s), including 1 subject who demonstrated broad BL ST reactivity to cephalosporins and penicillins (Table III). All 5 subjects had a history of anaphylaxis to cefazolin. One cefazolin-allergic patient with a positive ampicillin ST result in fact tolerated a penicillin VK OPC; the other 4 were not challenged. To date, 27 of the 44 subjects with negative penicillin ST results have undergone a penicillin OPC and, as expected, all were tolerated.

**Table III:** Positive ST/IDT results demonstrating non-R1 side-chain BL cross-/co-reactivity among subjects with confirmed index cephalosporin allergies, and no preexisting penicillin allergy

Subject	Index culprit drug (index reaction)	Preexisting PAL	Positive ST/IDT results	OPC
1	Cefazolin (grade 4 reaction; <1 y earlier)	Nil	Cefazolin +++, benzylpenicillin ++, ampicillin +++	Not performed
2	Cefazolin (grade 4 reaction; timing unknown)	Nil	Cefazolin ++, benpenzylpenicillin +, amoxicillin ++, ampicillin +	Not performed
3	Cefazolin (grade 4 reaction 1-5 y earlier)	Nil	Cefazolin +++, ampicillin +++	Tolerated penicillin VK
4	Cefazolin (grade 4 reaction; >5 y earlier)	Nil	Cefazolin +++, amoxicillin +++	Not performed
5	Cefazolin (grade 3 reaction; <1 y earlier)	Nil	Cefazolin,∗ MDM ++, PPL +, benpenzylpenicillin ++, amoxicillin +, ampicillin +, cefalexin +, ceftriaxone +, cefepime +	Not performed

The ST result was considered positive if the wheal diameter was at least 3 mm larger than that achieved with the negative control with surrounding erythema (flare). The IDT result was considered positive if the wheal diameter was at least 3 mm greater, and erythema was at least 5 mm greater, than that achieved with the control wheal (saline bleb). Wheal size summarized as 3 mm +, 4-7 mm ++, ≥ 8 mm +++.

*IDT*, Intradermal test; *MDM*, minor determinant mixture; *PPL*, penicilloyl polylysine.

∗ Cefazolin IDT performed in anesthetics drug allergy clinic, documented clear positive; however, wheal/flare size not documented.

In the remaining cohort, another 18 (8.5%) subjects were confirmed allergic to an R1-related antibiotic (aminopenicillins in 17 of 18) where a true index CAL was clinically suspected but not definitively confirmed. Thirty (14.2%) subjects were proven nonallergic to the index cephalosporin by OPC. Seventeen of these 30 subjects had PALs; however, none were confirmed by testing.

There were 14 subjects who reported multiple CALs (5 involving R1-related cephalosporins, 9 involving R1-unrelated cephalosporins) and all 14 also reported PALs. One CAL was confirmed (cefazolin ST+), and another subject had positive ST result to multiple BLs, suggesting a possible BL ring allergy. Eight of the 14 subjects underwent a penicillin OPC, of which 3 (37.5%) subjects reacted—none of whom had confirmed CALs.

This study presents a real-world analysis of test outcomes in cephalosporin- allergic subjects. We found that 27% of patients had their cephalosporin allergy confirmed, and that a history of anaphylaxis during the index reaction and an intravenous culprit drug were statistically significant predictors of a positive index or R1-related cephalosporin ST result. This finding is consistent with previous literature.^8^^,^^13^

Our major finding is that subjects with a confirmed cephalosporin allergy may demonstrate cross-reactivity within the R1 side-chain groups, but there was no evidence of broader cephalosporin group cross-reactivity.^7^^,^^14^ This finding reinforces the clinical relevance of the R1 side-chain model in cephalosporin allergy. The R1 side-chain model was similarly relevant to subjects with preexisting PALs, including examples where aminopenicillin/aminocephalosporin R1 side-chain cross-reactivity was confirmed, but a broader penicillin allergy was refuted.

It is well established that around 90% of patients with PALs (without CALs) can be delabelled after drug allergy assessment.^15^^,^^16^ Similarly, in our study, most subjects with preexisting PALs in fact tolerated a penicillin challenge, regardless of whether they had a true CAL. A notable exception was the higher penicillin OPC reaction rate in the 14 subjects with 3 or more BL allergy labels: 1 demonstrated broad BL ST positivity, and 3 of 8 reacted to a penicillin OPC. This finding suggests that clinicians should exercise more caution in assessing patients with multiple BL allergy labels, such as by avoiding direct challenges, without preceding skin tests.

The unexpected finding in this study was the penicillin ST reactivity observed in 5 (10.2%) of the confirmed cefazolin-allergic subjects. We note, however, that there were no examples of penicillin allergy demonstrated by OPC in the cephalosporin-allergic patients without PALs. Four of the 5 cefazolin and penicillin ST-positive subjects did not undergo a follow-up penicillin challenge due to safety concerns and lack of clinical indication. Our findings are similar to those of previous reports, including a meta-analysis reporting that 3.7% to 4.4% of cefazolin-allergic patients have positive ST results to penicillins.^7^^,^^8^^,^^17^ However, a recent study highlighted instances of false-positive penicillin ST results in cefazolin-allergic patients, reporting that 5 of 68 (7.3%) subjects with positive cefazolin ST results also had positive penicillin ST results, but in fact went on to tolerate a penicillin challenge.^18^ The ST results in our cohort of cefazolin-allergic patients demonstrated significant wheal and flare, with multiple positive ST results in 3 cases, making false-positive results less likely. Ultimately, without follow-up penicillin challenges, it is not possible to determine whether these penicillin ST results represent false positives, true cross-reactivity, or cosensitization to penicillins.

The limitations of this study are the retrospective data collection, incomplete testing and inconclusive test outcomes in a number of cases, limited number of subjects with confirmed allergies to noncefazolin cephalosporins, and lack of confirmatory challenges in subjects with positive ST results. We acknowledge that some of the intradermal skin test concentrations used in this study differ from those used in some other centers; however, ST concentrations used were contemporary to guidelines available at the time this study commenced. Repeat testing to assess for resensitization following negative drug allergy workup was not performed, given lack of global consensus for this approach, and resource constraints.^10^^,^^19^

### Conclusion

Cross-reactivity between cephalosporins, and penicillins, is mainly explained by R1 side-chain similarity, with infrequent examples of broad BL cross-reactivity. However, some positive penicillin ST results do not clearly follow these patterns, and the presence or absence of a PAL does not reliably predict penicillin test results in cephalosporin-allergic patients. Future research investigating the safety of supervised graded penicillin challenges in cephalosporin-allergic patients is needed.

**Clinical implications**

Cross-reactivity between cephalosporins, and penicillins, is mainly explained by R1 side-chain similarity, but some positive ST results do not follow this pattern. The presence of a preexisting PAL is not a good predictor of penicillin skin test or OPC outcomes in cephalosporin-allergic patients.

## Disclosure statement

Disclosure of potential conflict of interest: The authors declare that they have no relevant conflicts of interest.
